# Brain creatine metabolism modulated by hydrogen-enriched water

**DOI:** 10.1080/15502783.2025.2533654

**Published:** 2025-07-21

**Authors:** Dragana Zanini, Nikola Todorovic, Korovljev Darinka, Stajer Valdemar, Bogdan Andjelic, Marijana Ranisavljev, Jovan Kuzmanovic, Sergej M. Ostojic

**Affiliations:** a Applied Bioenergetics Lab, Faculty of Sport and Physical Education, University of Novi Sad, Novi Sad, Serbia; b Faculty of Health Sciences, University of Pécs, Pécs, Hungary; c Department of Nutrition and Public Health, University of Agder, Kristiansand, Norway

**Keywords:** Creatine, brain, magnetic resonance spectroscopy, hydrogen, dietary supplements, clinical trial, gray matter, white matter

## Abstract

**Background:**

Creatine (Cr) plays a crucial role in aging by supporting cognitive function and promoting neuroprotection. Optimizing Cr levels in the aging brain is therefore of significant interest. This study aimed to explore whether hydrogen-rich water (HRW) supplementation could influence brain creatine metabolism in older adults.

**Methods:**

In a double-blind, randomized, placebo-controlled pilot trial, eight healthy individuals over 70 years of age (mean age: 75.6 ± 5.2 years; 4 females) underwent magnetic resonance spectroscopy (MRS) before and after six months of daily consumption of 500 mL of HRW or a placebo. Creatine levels were assessed at 13 brain regions using single-voxel spectroscopy for the left thalamus and a 2D multi-voxel PRESS technique for 12 additional regions, covering frontal, middle, and posterior white and gray matter bilaterally.

**Results:**

Participants in the HRW group exhibited significant creatine changes in specific brain regions after six months. Compared to the placebo group, HRW supplementation was associated with increased creatine levels in the right parietal white matter (from 6.0 ± 1.1 to 6.3 ± 1.6 ppm) and decreased creatine levels in the left parietal-mesial gray matter (from 4.4 ± 2.3 to 4.2 ± 2.3 ppm) (P < 0.05). Additionally, a trend toward increased creatine levels was observed in the right frontal gray matter in the HRW group (from 3.8 ± 2.0 to 5.0 ± 1.8 ppm) compared to the placebo (from 4.7 ± 0.9 to 4.5 ± 0.9 ppm) (P = 0.08).

**Conclusion:**

In addition to conventional creatine-enhancing supplements, hydrogen-based supplementation may modulate age-related and region-specific creatine dynamics in the brain. Further research with a larger cohort is needed to validate these findings.

